# Supplementary material for: The Construction and Exploration of a Comprehensive MicroRNA Centered Regulatory Network in Foxtail Millet (Setaria italica L.)
Source: Front Plant Sci. 2022 May 6;13:848474. doi: 10.3389/fpls.2022.848474 (PMC9121102; doi:10.3389/fpls.2022.848474)
Supplement: Supplementary file 6 [file Data_Sheet_6.zip › Sit-miR3979_Seita.7G294400.1_535_TPlot.pdf]

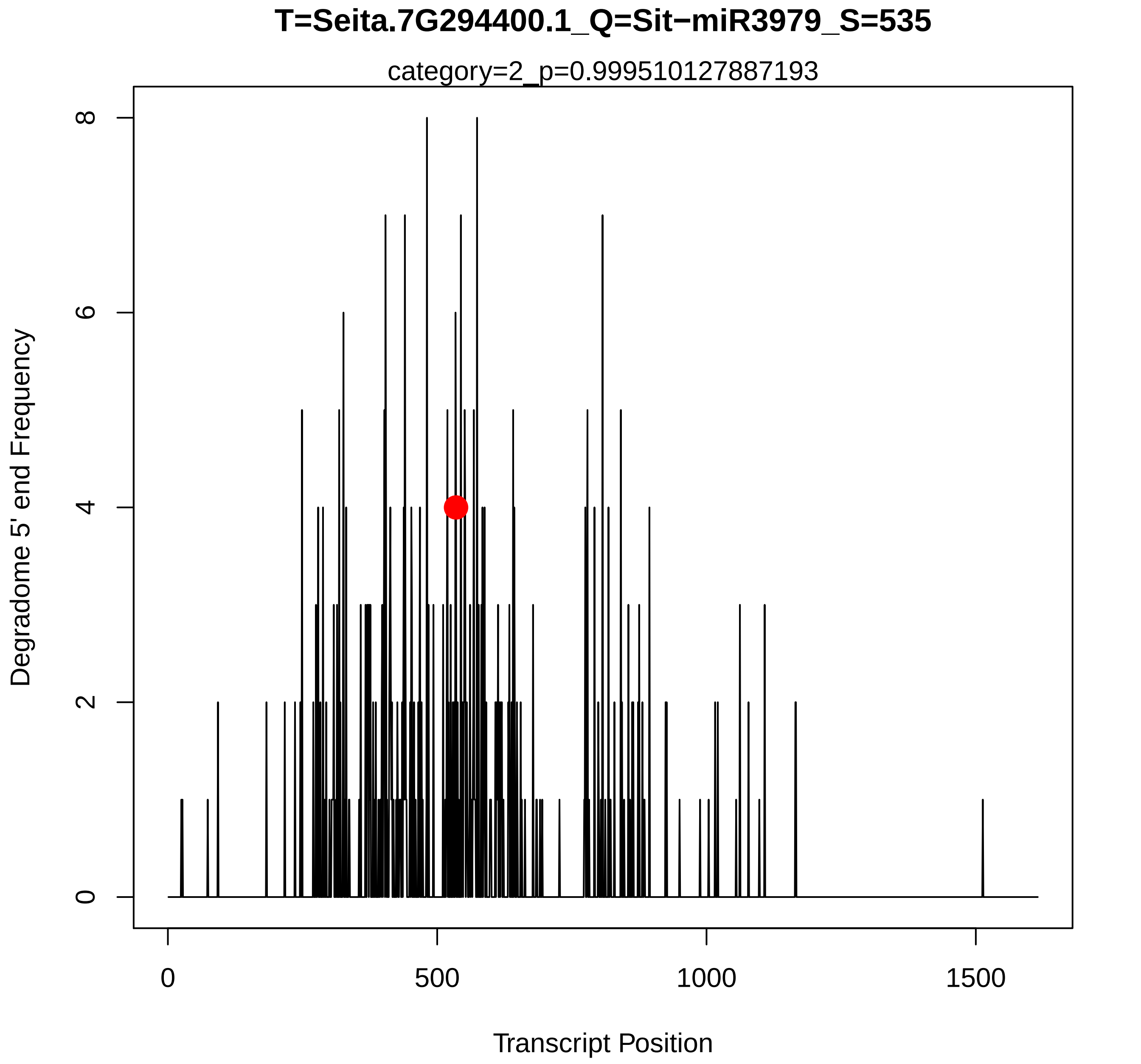

T=Seita.7G294400.1_Q=Sit−miR3979_S=535
category=2_p=0.999510127887193
Degradome 5' end Frequency
0
2
4
6
8
0
500
1000
1500
Transcript Position
